# Maternally inherited coronary heart disease is associated with a novel mitochondrial tRNA mutation

**DOI:** 10.1186/s12872-019-01284-4

**Published:** 2019-12-16

**Authors:** Zhenxiao Zhang, Mingyang Liu, Jianshuai He, Xiaotian Zhang, Yuehua Chen, Hui Li

**Affiliations:** 1 Department of Emergency, Affiliated hospital of Qingdao university, Jiangsu Road No. 16, Qingdao, 266000 Shandong China; 2 Department of Anesthesiology, Affiliated hospital of Qingdao university, Qingdao, 266000 Shandong China; 3 Department of ICU, Affiliated hospital of Qingdao university, Qingdao, 266000 Shandong China

**Keywords:** Coronary heart disease (CHD), Mitochondrial tRNA, Mutation, Chinese

## Abstract

**Background:**

Coronary heart disease (CHD) is the most common cause of mortality globally, yet mitochondrial genetic mutations associated with CHD development remain incompletely understood.

**Methods:**

The subjects from three Chinese families with LHON underwent clinical, genetic, molecular, and biochemical evaluations. Biochemical characterizations included measuring the effects of the15910C > T mutation on tRNA^Thr^ levels, enzymatic activity of electron transport chain complexes, membrane permeability, and the mitochondria-mediated generation of both reactive oxygen species (ROS) and adenosine triphosphate (ATP).

**Results:**

We characterize mitochondrial genetic mutations in a three-generation Chinese family exhibiting signs of maternally inherited CHD. Of the 24 different family members in this pedigree we assessed, CHD was detected in 6, with variable severity and age of first appearance. When we sequenced the mitochondrial genomes of these individuals, we found a tRNA^Thr^ 15910C > T mutation of the Eastern Asian haplogroup M7b’c. This mutation is predicted to destabilize the strongly conserved (24C-10G) base-pairing, thereby disrupting tRNA^Thr^ functionality. When we performed Northern blotting, we detected we observed a 37.5% reduction in tRNA^Thr^ levels at baseline in cybrid cell lines bearing the 15910C > T mutation. When we conducted western blot analysis, we detected a ~ 24.96% decrease in mitochondrial translation rates in these same cells.

**Conclusions:**

In the present report, Together these findings suggest a possible link between this 15910C > T tRNA^Thr^ mutation and CHD, potentially offering new avenues for future disease intervention.

## Background

Different Cardiovascular diseases remain the most prominent cause of death globally, with coronary heart disease (CHD) remaining a highly complex and heterogeneous disease the onset of which is typically influenced by a range of environmental and genetic factors, although in some cases single gene mutations can drive disease [1, 2]. While many studies to date have sought to identify nuclear genomic factors linked with CHD incidence, relatively few studies have specifically investigated CHD risk arising as a consequence of mitochondrial mutations [3–5]. Such investigations are important, as abnormal mitochondrial functionality has been found to be a potential driver of hypertension and other cardiovascular diseases [6, 7]. Multiple previous studies have identified linked between specific mitochondrial DNA (mtDNA) mutations and hypertension, including the tRNA^Ile^ 4295A > G mutation, as well as the tRNA^Ile^ 4263A > G, tRNA^Met^/tRNA^Gln^ 4401A > G, and tRNA^Met^ 4435A > G mutations which were specifically linked to hypertension in Chinese individuals [8–11].

To explore additional mutations linked with CHD pathogenesis, we are conducting ongoing systematic screening efforts assessing mtDNA mutations among Chinese CHD patients. As a part of this effort, we have identified one three-generation family presenting with evidence of CHD transmitted matrilineal, with 6/24 analyzed adults in this family exhibiting CHD of varying severity. When we analyzed their mtDNA, we detected the presence of a tRNA^Thr^ 15910C > T mutation of the M7b’c haplogroup. This mutation occurred in the stem region of this tRNA (conventional position 25) a site that is highly conserved and the mutation of which is predicted to result in structural and functional changes that have the potential to disrupt normal mitochondrial functionality. After identifying this mutation we followed up on its biological significance in cybrid cell lines bearing this mutation, measuring the effects of this15910C > T mutation on tRNA^Thr^ levels, enzymatic activity of electron transport chain complexes, membrane permeability, and the mitochondria-mediated generation of both reactive oxygen species (ROS) and adenosine triphosphate (ATP).

## Methods

### Subjects

Eighty genetically unrelated Han Chinese subjects with CHD including a three-generation Han Chinese family (Q5) were recruited from affiliated hospital of Qingdao university. In addition, 113 unrelated controls were also obtained from among volunteers in the same area. All participants provided informed consent, and underwent both clinical evaluation and blood sample collection. The Qingdao University ethics committee oversaw and approved this study.

### Assessment of risk factors

Relevant risk factors in the present study included hypertension, hyperlipidemia, diabetes, a history of smoking, or a family history of CHD. Patient blood pressure was measured according to standard methods using the average of three readings. Hypertension was designated as a systolic blood pressure ≥ 140 mmHg and/or a diastolic blood pressure ≥ 90 mmHg as per JNC VI criteria. Diabetes was diagnoses in patients based on the presence of either a need for antidiabetic medications, or fasting blood glucose > 126 g/dL, as in previous reports.Any individuals who reported having used cigarettes within the past 12 months were designated smokers.

### Mitochondrial mutational assessment

The Puregene DNA Isolation Kits (Biomega) was used to isolate total genomic DNA from study participants, after which mitochondrial genomic DNA was assessed via Southern blotting as in previous research. A total of 24 overlapping PCR fragments were generated and amplified in order to provide full coverage of the mitochondrial genome, using appropriate pairs of light/heavy strand primers used in previous studies. An ABI 3700 automated DNA sequencer was then employed to sequence each of these fragments following purification with a Big Dye Terminator Cycle sequencing reaction kit. The consensus revised Cambridge sequence (GenBank accession number: NC_012920) was then used for alignment of these sequenced fragments. Detection of the 15910C > T mutation in family members, 80 unrelated CHD patients, and other controls was performed as in previous studies.

### Cell culture

The Epstein-Barr virus was used to generate immortalized patient cell lines from the proband patient (III-3) bearing the 15910C > T mutation, as well as from a control individual (C2). These cells were cultured in RPMI 1640 containing 10% FBS. Cybrid cells were generated by adapting previous protocols. Briefly, bromodeoxyuridine (BrdU)-resistant 143B.TK^−^ cells were cultured in DMEM containing 5% FBS, and the ρ^o^206 cell line lacking mtDNA derived from these same cells was also grown under these conditions in the presence of 50 μg uridine/ml. The patient and control cell lines were then enucleated and fused with the ρ^o^ 206 cells. The resultant cybrid cell lines were then selected in uridine-free DMEM supplemented with BrdU, allowing for donor-derived cybrid lines that could then be assessed for the m.15910C > T mutation, amounts of mtDNA, and other cellular genetic features. The resultant cybrid lines were maintained in DMEM containing 5% FBS.

### Northern blotting

TRIzol was used to isolate total mitochondrial RNA from knockdown or control cell lines, as in previous studies [12]. In brief, two micrograms of total mitochondrial RNA were electrophoresed through a 10% polyacrylamide/7 M urea gel in Tris–borate–Ethylenediaminetetraacetic acid (EDTA) buffer (TBE) (after heating the sample at 65 °C for 10 min), and then electroblotted onto a positively charged nylon membrane for the hybridization. Analysis with oligodeoxynucleotide probes. In total, we followed by transfer onto a positively charged membrane which was then combined with appropriate DIG oligodeoxynucleoside probes based on previously described approaches, using tRNA^Thr^, tRNA^His^, tRNA^Ala^, and tRNA^Glu^ utilized in previous studies [3, 12].

### Western blotting

Western blotting was used to assess protein levels in cells, via first electrophoretically separating 20 μg of protein from each sample via SDS-PAGE. These samples were then transferred onto PVDF membranes, which were then probed with primary antibodies against ND4, ND1, ND6, CYTB, ATP6, CO2, and VDAC. Secondary Affini Pure goat anti-mouse IgG and goat anti-rabbit IgG conjugated to peroxidase enzymes were then used to probe these blots, followed by use of an ECL system for chemiluminescent detection. Densitometric band quantification was then performed as in previous studies [9, 10].

### Measurements of enzymatic activity

The complex I, II, III, and IV activities were assessed as in previous studies [12]. In brief, Citrate synthase activity was analyzed by the reduction of DTNB at 412 nm in the assay buffer. Complex I activity was determined by following the decrease in the absorbance due to the NADH oxidation at 340 nm in assay buffer. The activity of complex II was analyzed by tracking the secondary reduction of 2,6-dichlorophenolindophenol by decylubiquinone (DB) at 600 nm in the assay buffer. Complex III activity was determined by measuring the reduction of cytochrome c at 550 nm with reduced decylubiquinone in the assay buffer. Complex IV activity was measured by monitoring the oxidation of reduced cytochrome c as a decrease of absorbance at 550 nm. Complex I-IV activities were normalized by citrate synthase activity [3, 12].

### Measuring ATP levels

In order to assess ATP generation in cells, the Cell Titer-Glo Luminescent Cell Viability Assay kit (Promega) was used based on provided protocols [12]. In brief, after a 30 min equilibration of the cell plate to room temperature, 100 μl of the assay reagent was added into each well with 20,000 cells and the content was mixed for 2 min on an orbital shaker to induce cell lysis. After 10 min incubation in room temperature, the luminescence was read on a microplate reader [12].

### Mitochondrial membrane potential measurements

The JC-10 Assay Kit (Abcam) was used to measure mitochondrial membrane potential based on provided protocols as detailed elsewhere [3, 12]. In brief, cells were plated onto 96-well cell culture plate overnight in growth medium. In the first plated cell, JC-10 dyeloading solution was added for 30 min at 37 °C, 5% CO_2_. Alternatively, the second plated cells were preincubated with 10 μM of the FCCP for 30 min at 37 °C, 5% CO_2_ prior to staining with JC-10 dye. Samples were measured at Ex/Em = 490/530 and 490/590 nm with a microplate reader [3, 12].

### Assessment of ROS levels

The MitoSOX Red Mitochondrial Superoxide Indicator (Invitrogen, M36008) was used to assess ROS production in live cells based on provided protocols as described previously [3, 12]. In brief, approximately 2 × 10^6^ cells of each cell line were harvested, resuspended in PBS, supplemented with 100 μM of MitoSOX and 2% FBS. After incubation at 37 °C for 20 min. Finally, cells were resuspended. Samples were analyzed by the flow cytometer system, with an excitation at 488 nm and emission at 529 nm. 10,000 events were analyzed in each sample [3, 12].

### Statistical analysis

Unpaired, two-tailed Student’s t-tests were used to compare all values in this study. SPSS v17.0 and GraphPad Prism v 6.0 were used for all analysis, and *p* < 0.05 was the significance threshold.

## Results

### Clinical presentation

**Fig. 1:**
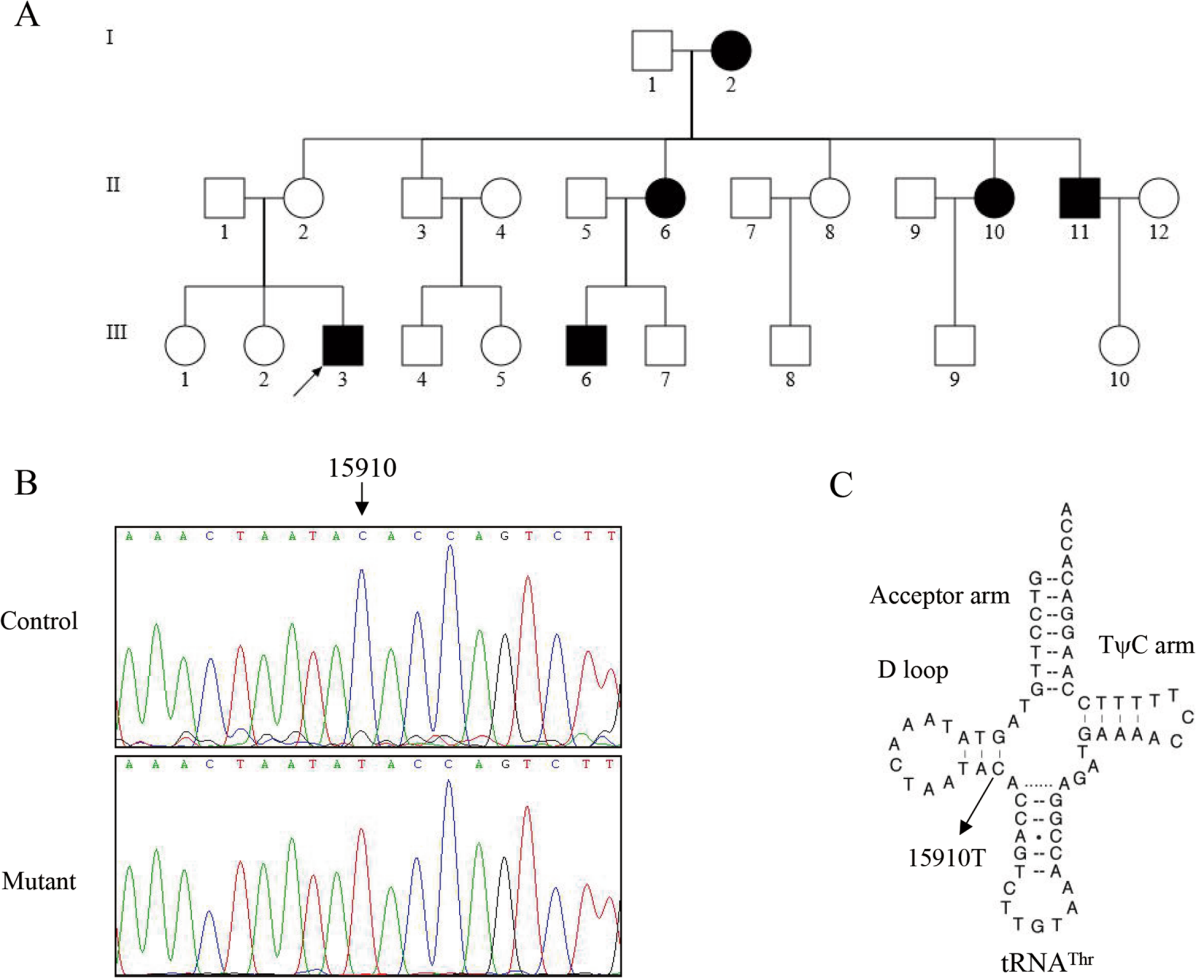
The Chinese pedigree with CHD. **a** Vision-impaired individuals are indicated by blackened symbols. **b** Identification of the 15910C > T mutation in the tRNA gene. Partial sequences chromatograms of tRNA gene from the proband and one Chinese control. **c** The location of the 15910C > T mutation in the mitochondrial tRNA^Thr^. An arrow indicates the location of the base changes at position 15,910

The proband (Q5-III-3) was first diagnosed with CHD upon presenting at the Cardiology Clinic of affiliated hospital of Qingdao university, after which he underwent a full medical evaluation. The patient was diagnosed with hypertension (159/99 mmHg), significant ischemia (65% narrowing was evident upon coronary angiography), and high cholesterol (LDL-C = 159 mg/dL, TC = 232 mg/dL). The patient was not affected by any other comorbid conditions such as diabetes or neurological disease. When family members of this patients were evaluated for these same conditions, 5 were diagnosed with all three of these conditions (Fig. 1a and Table 1). In each case, any fathers with CHD had not transmitted it to their children, whereas mothers did transmit it, suggesting matrilineal inheritance consistent with mitochondrial involvement in this inherited CHD risk.

**Table 1:** Summary of clinical data for some members in a Chinese pedigree

Subjects	Gender	Age of onset (years)	Systolic pressure/Diastolic pressure (mmHg)	ECG	Ectent of CAD narrow (%)	total cholestero (mg/ dl)	LDL (mg/ dl)
II-6	F	60~65	150/95	ischemia	55	190	140
II-10	F	55~60	140/90	ischemia	50	220	145
II-11	M	50~55	138/85	ischemia	50	210	150
III-3	M	40~45	159/99	ischemia	65	232	159
III-6	M	35~40	145/90	ischemia	50	187	140

### Analysis of mitochondrial mutations

**Fig. 2:**
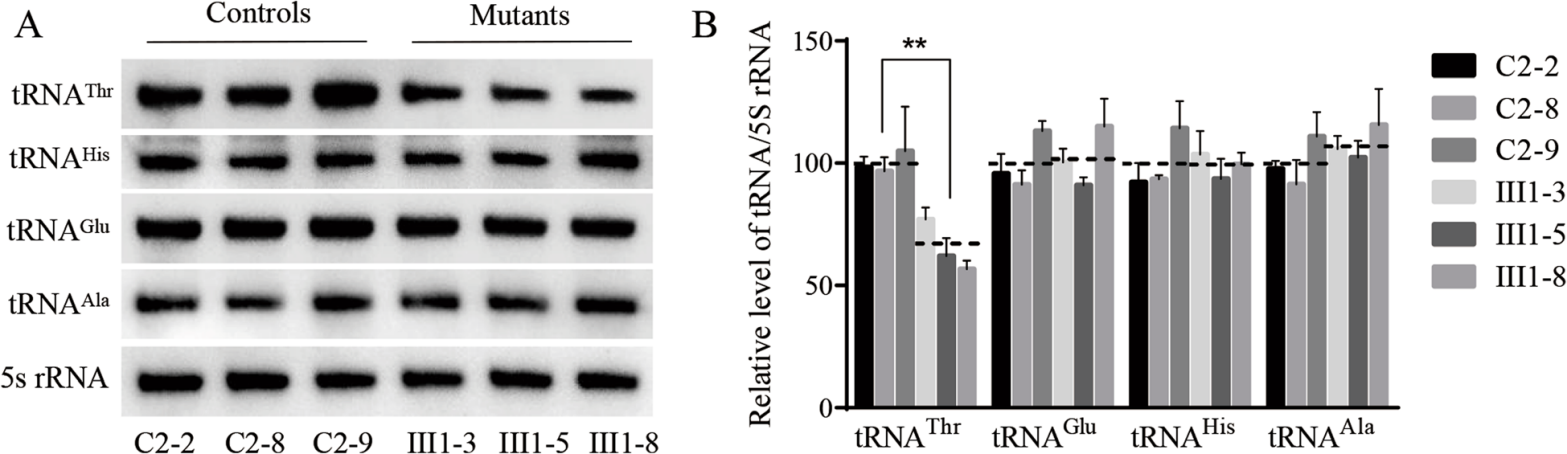
Northern blot analysis of mitochondrial tRNA. **a** Equal amounts of total mitochondrial RNA from various cell lines were electrophoresed through a denaturing polyacrylamide gel, electroblotted and hybridized with DIG-labeled oligonucleotide probes specific for the tRNA^Thr^, tRNA^His^, tRNA^Glu^, tRNA^Ala^ respectively. **b** Quantification of tRNA levels. Average relative tRNA content per cell, was normalized to the average content per cell of 5S rRNA in three mutant cybrid cell lines(III1–3, III1–5 and III1–8) carrying the 15910C > T and control cybrid cell lines (C2–2, C2–8 and C2–9). The values for the latter are expressed as percentages of the average values for the control cell lines

To explore potential mitochondrial mutations linked with inherited CHD risk, we sequenced the mitochondrial genome of this proband patient Q5-III-3. A total of 45 mutations were evident in their mitochondrial genome upon comparison with the revised Cambridge consensus sequence (NC_012920), and the mitochondrial haplogroup for this patient was identified to be M7b’c (Fig. 2). As shown in Table 2, of these 45 variants, 19 were known silent variants, 14 were known D-loop variants, 8 were known missense mutations affecting protein-coding genes, 2 were known 12S rRNA variants, 1 was a known 16 s rRNA variant, and one was a novel homoplasmy 15910C > T mutation in the tRNA^Thr^ gene (Fig. 1b). The detected missense mutations were as follows: 5460G > A (Ala331Thr) in the ND2 gene, 7853G > A (Val90Ile) in the CO2 gene, the 8701A > G (Thr59Ala) in the ATP6 gene, the 10398A > G (Thr114Ala) in the ND3 gene, 12,811 T > C (Tyr159His) in the ND5 gene, the 14766C > T (Thr > Ile), 14978A > G (Ile78Val), and m.15326A > G (Thr > Ala) in the CYTB gene. We compared the variance at these mutated RNA residues phylogenetically across 16 different primate species, revealing this tRNA^Thr^ 15910C > T mutation to have a 100% conservation index across species, making it more likely to have functional significance when mutated as in this patient. Moreover, this mutation was also not detected when other 79 unrelated patients and 113 Chinese control subjects were analyzed.

**Table 2:** mtDNA variants in a Chinese family with CHD

Gene	Position	Replacement	AA change	Conservation	Previously reported
D-loop	73	A-G			Yes
	143	G-A			Yes
	150	C-T			Yes
	199	T-C			Yes
	204	T-C			Yes
	207	G-A			Yes
	263	A-G			Yes
	310	T-C			Yes
	489	T-C			Yes
	16,129	G-A			Yes
	16,189	T-C			Yes
	16,223	C-T			Yes
	16,248	C-T			Yes
	16,297	T-C			Yes
12SrRNA	750	A-G			Yes
	1438	A-G			Yes
16SrRNA	2706	A-G			Yes
ND1	4071	C-T			Yes
	4164	A-G			Yes
ND2	4679	T-C			Yes
	4769	A-G			Yes
	5351	A-G			Yes
	5460	G-A	Ala331Thr	5.88%	Yes
CO1	6455	C-T			Yes
	6680	T-C			Yes
	7028	C-T			Yes
CO2	7684	T-C			Yes
	7853	G-A	Val90Ile	29.41%	Yes
ATP6	8701	A-G	Thr59Ala	52.94%	Yes
CO3	9540	T-C			Yes
	9824	T-C			Yes
ND3	10,398	A-G	Thr114Ala	41.18%	Yes
	10,400	C-T			Yes
ND4	10,873	T-C			Yes
	11,719	G-A			Yes
ND5	12,405	C-T			Yes
	12,705	C-T			Yes
	12,811	T-C	Tyr159His	64.71%	Yes
Cytb	14,766	C-T	Thr7Ile	47.06%	Yes
	14,783	T-C			Yes
	14,978	A-G	Ile78Val	47.06%	Yes
	15,043	G-A			Yes
	15,301	G-A			Yes
	15,326	A-G	Thr194Ala	52.94%	Yes
tRNA^Thr^	15,910	C-T		100%	No

As presented in online mitochondrial genome databases: www.mitomap.org and www.genpat.uu.se/mtDB

### Mutation leads to decreased mitochondrial tRNA^Thr^ levels

We next assessed how this 15910C > T mutation altered the metabolism of tRNA^Thr^, subjecting cybrid cell lines bearing this mitochondrial mutation to Northern blotting using probes specific to this and 3 other tRNAs. We found that tRNA^Thr^ levels in these mutant cybrid lines were significantly reduced relative to control wild type cells (Fig. 2), with baseline tRNA^Thr^ levels in these mutant cells being 65.25% of those in control cells, with 5S RNA used for normalization purposes. In contrast, baseline tRNA^His^, tRNA^Ala^, and tRNA^Glu^ levels in these mutant cell lines were unchanged relative to control cells (102.13, 98.89, and 107.91%, respectively).

### Mutation leads to reduced mitochondrial protein levels

**Fig. 3:**
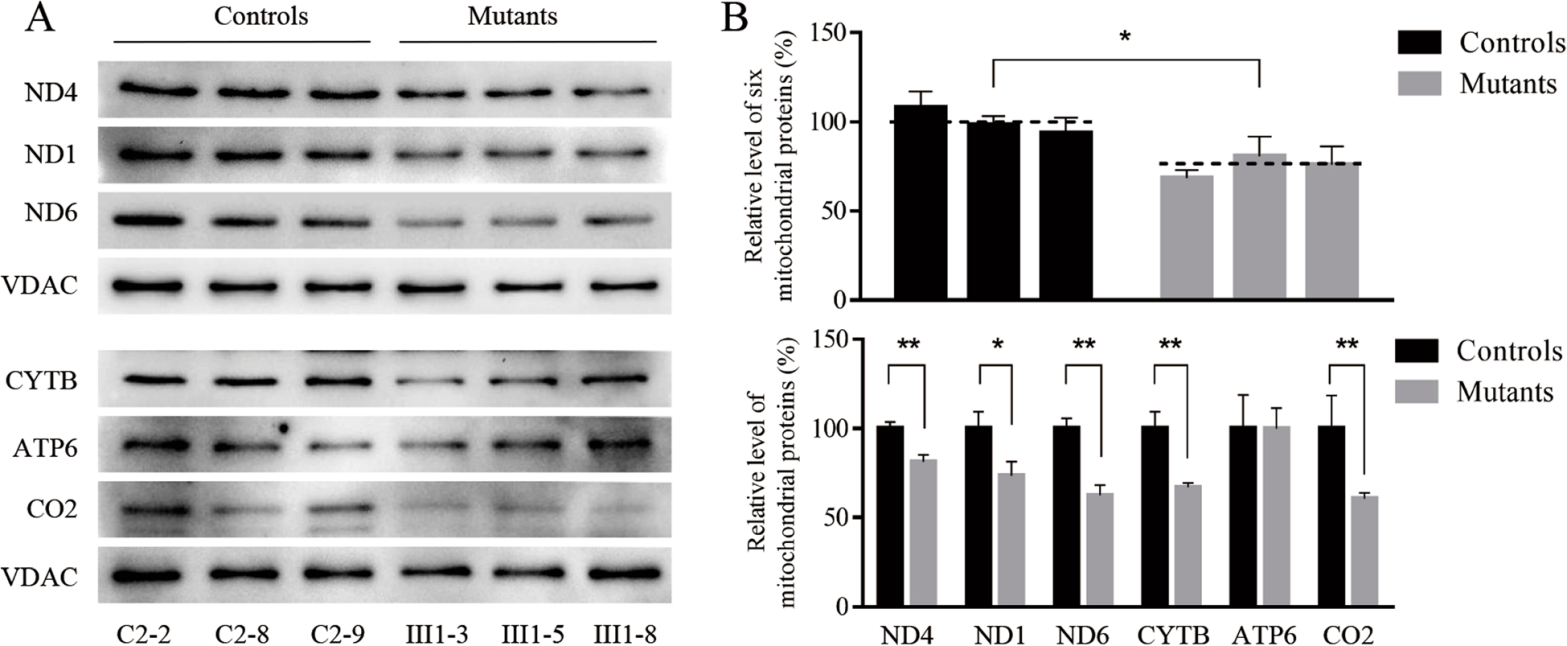
Western blot analysis of mitochondrial proteins. **a** Twenty micrograms of total cellular proteins from various cell lines were electrophoresed through a denaturing polyacrylamide gel, electroblotted and hybridized with respiratory complex subunits in mutant and control cells with VDAC as a loading control. **b** Quantification of 6 respiratory complex subunits. The levels of ND6, ND4, ATP6, CYTB, CO2 and ND1 in three mutant cybrid cell lines and control cybrid cell lines were determined. The error bars indicate two standard errors of the means. *p* indicates the significance, according to the *t*-test, of the differences between mutant and control cell lines

We next performed Western blotting to assess levels of the mtDNA-encoded components of the respiratory complex in cells bearing the 15910C > T mutation or controls. As shown in Fig. 3, e found that mutant cells expressed mitochondrial protein levels that were 19.31 to 31.55% of those in control cells (average was 24.96%; *P* < 0.05). These mutated cells also showed clear reductions (18.71, 26.56, 37.52, 33.00 and 39.48%) in 5 polypeptides (ND4, ND1, ND6, CYTB and ATP6), while CO2 levels were not significantly reduced (0.15%) relative to control cells.

### Mutation led to decreased complex I and III activity

**Fig. 4:**
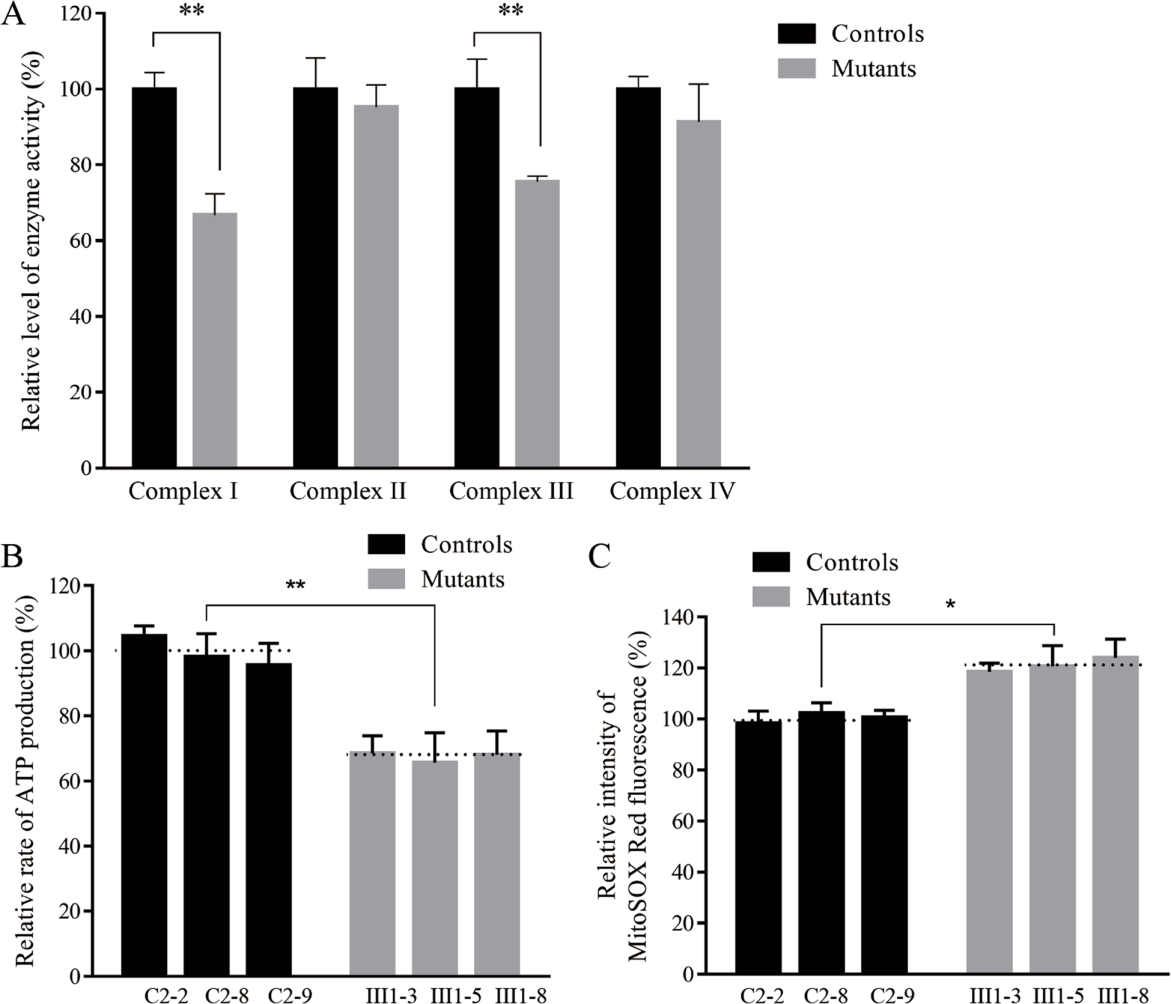
Measurement of cellular in mitochondria**. a** Respiratory complex activities. The activities of respiratory complexes were investigated by enzymatic assay on complexes I, II, III, and IV in isolated mitochondria from lymphoblastoid cell lines derived from the mutant and control cybrid cell lines. Activities of complexes I, II, III, and IV were normalized by citrate synthase activity. **b** mitochondrial ATP levels. Mutant and control cell lines were incubated with 10 mM glucose or 5 mM 2-deoxy-d-glucose plus 5 mM pyruvate to determine ATP generation under mitochondrial ATP synthesis. Average rates of ATP level per cell line in mitochondria are show. The determinations were made for each cell line.The calculations were based on the independent determinations in each cell line. **c** Ratio of geometric mean intensity. Measurement of mitoROS. The levels of ROS generation by mitochondria in living cells from mutant and control cell lines were determined using the mitochondrial superoxide indicator MitoSOX-Red. The average of the determinations for each cell line is shown. The error bars indicate two standard errors of the means. *p* indicates the significance, according to the *t*-test, of the differences between mutant and control cell lines

We further assessed the consequences of this m.15910C > T mutation on oxidative phosphorylation using isolated mitochondrial from our mutant and control cybrid cell lines. We found that complex I and III activity in the 15910C > T mutant mitochondria was 66.72, and 75.48% that of the activity observed in control cells, whereas no changes in complex II/IV activity were observed(Fig. 4a).

### Mutation leads to reduced mitochondrial ATP generation

We further assessed the generation of ATP by wild type and mutant cells in an effort to better gauge how this mutation influenced oxidative phosphorylation. To test this, either glycolysis or oxidative phosphorylation were selectively induced in cells via culture with glucose, glucose + oligomycin, or 2deoxy-D-glucose + pyruvate. When cells could only engage in oxidative phosphorylation, mutant cells bearing the 15910C > T mutation,exhibited ATP production that was 65.68–67.98% (average: 67.37%) of that in control cells(Fig. 4b).

### Mutation leads to increased ROS production

We next assessed ROS production in our mutant cybrid cell lines via flow cytometry, comparing baseline staining intensity for each cell line to that upon oxidative stress in order to obtain a ratio corresponding to ROS generation. We observed somewhat increased ROS generation for our mutant cybrid cell lines bearing the 15910C > T mutation, with ROS production 118.45–123.98%, (average: 121.04%) that of control cells (Fig. 4c).

## Discussion

Mitochondrial DNA mutations have been associated with CHD in precious studies [13–15]. In this study, we offer evidence of a novel mitochondrial mutation which is linked to an elevated risk of CHD. This mutation was detected among adult matrilineally-related individuals in a three-generation Chinese family, with affected individuals presenting with CHD, hypertension and hyperlipidemia. This CHD risk was matrilineally inherited, and a mutational analysis revealed the presence of a 15910C > T mutation at the C25 position in the tRNA^Thr^ sequence - a residue which is normally highly conserved and which is predicted to be important for tRNA stability(Fig. 1c). This mutations is predicted to destabilize the base-pairing at this site (25C-10G), potentially altering the secondary structure of this tRNA, as has previously been reported for the tRNA^Ile^ 4300A > G and tRNA^Leu(UUR)^ 3273 T > C mutations [16, 17].

When cybrid cells bearing this mutation were generated, their baseline tRNA^Thr^ levels were reduced by 37.5% relative to healthy control cells, suggesting that there may be a resultant destabilization of this mutated tRNA^Thr^ resulting in its more rapid degradation, as previously described previously such as the 3243A > G mutation of tRNA^Leu(UUR)^ [18–21]. As the mitochondrial dysfunction stemming from the 15910C > T mutation was relatively mild, this suggests that this mutation alone is unlikely to cause CHD. As shown in Additional file 1: Table S1, we observed a ~ 24.96% reduction in mitochondrial protein levels in cells bearing this mutation, and these cells als exhibited altered complex I/III activity which may coincide with increased electron leakage and elevated ROS production. In the present study, the reduced levels of mitochondrial proteins (an average decrease of ~ 29%) were comparable with the reduced rate of mitochondrial protein synthesis observed in cell lines bearing the hypertension-associated m.4435A > G, m.4401A > G and m.4263A > G mutations [9–11]. Indeed, consistent with this we found that ROS production was elevated in cybrids expressing this 15910C > T mutation. Such ROS production can lead to significant damage to cellular macromolecules including DNA and proteins, potentially leading to cellular dysfunction or apoptotic cell death which, if it were to occur in cardiac muscle cells, could contribute to the observed CHD phenotype, potnetially explaining how these mutations contribute to the observed matrilineal CHD, as hypertension-associated mitochondrial tRNA^Ala^ m.5655A > G m.5587 T > C, tRNA^Leu(CUN)^ m.12280A > G and tRNA^Met^ m.4467 C > A detailed previously [22–24].

As the 15910C > T mutation was homoplasmic in nature in the study subjects, this suggests that the mutation is relatively mild, consistent with the limited changes in mitochondrial functionality observed herein. Even so, our study suggests that this gene mutation is linked to an elevated risk of CHD development, with the ultimate odds of CHD development likely depending on a combination of environmental, lifestyle, and nuclear genetic factors in concert with the observed mitochondrial dysfunction. Indeed, other mutations in nuclear genes may also contribute to mitochondrial dysfunction in patients bearing the 15910C > T mutation, potentially resulting in the observed CHD phenotype. Moreover, the nuclear genetic or epigenetic factors may contribute to the development of clinical phenotype in subjects carrying the 15910C > T mutation.

The study has several limitations. Firstly, this investigation is limited by relatively low number of patients included because the subjects only obtained from one hospital. Secondly, the homoplasmic form, mild mitochondrial dysfunction, late onset, and incomplete penetrance of CHD observed in this Chinese family carrying the 15910C > T mutation suggest that the mutation is an inherited risk factor necessary for the development of CHD but may by itself be insufficient to produce a clinical phenotype. Finally, the tissue-specific effect of this 15910C > T mutation may be attributed to the tissue-specific RNA metabolism or the involvement of nuclear modifier genes.

## Conclusions

Our results suggest that the mitochondrial tRNA^Thr^ 15910C > T mutation is linked to CHD incidence. This mutation leads to altered metabolism of this particular tRNA, thus resulting in abnormal mitochondrial functionality and enhanced ROS production. Other nuclear genetic mutations may also act in concert with the 15910C > T mutation to amplify consequent mitochondrial dysfunction in affected patients, and extended ROS production in cardiovascular cells may be linked to CHD onset. Our results thus suggest a potential new mechanism liked to the underlying pathology of CHD, indicating future avenues for therapeutic research.

## Supplementary information

**Additional file 1: Table S1.** Summary of the mitochondrial functions in cybrid cell lines with control and mutant subjects.

## Data Availability

All data generated or analyzed during this study are included in this published article [and its supplementary information files]. Below are the links to the sequencing data generated in this study: https://www.ncbi.nlm.nih.gov/bioproject/PRJNA591378.

## Abbreviations

ATP: Adenosine triphosphate
CHD: Coronary heart disease
ROS: Reactive oxygen species
