# Supplementary material for: Maternally inherited coronary heart disease is associated with a novel mitochondrial tRNA mutation
Source: BMC Cardiovasc Disord. 2019 Dec 16;19:293. doi: 10.1186/s12872-019-01284-4 (PMC6912950; doi:10.1186/s12872-019-01284-4)
Supplement: Supplementary file 1 — Additional file 1: Table S1. Summary of the mitochondrial functions in cybrid cell lines with control and mutant subjects. [file 12872_2019_1284_MOESM1_ESM.doc]

Table S1. Summary of the mitochondrial functions in cybrid cell lines with control and mutant subjects.

| Mitochondrial function | control(%) | mutation(%) | *P* value |
| --- | --- | --- | --- |
| ATP | 100 | 67.37 | <0.001 |
| ROS | 100 | 121.04 | 0.005 |
| Enzyme activity |  |  |  |
| Complex I | 100 | 66.72 | <0.001 |
| Complex II | 100 | 95.21 | 0.342 |
| Complex III | 100 | 75.48 | 0.007 |
| Complex IV | 100 | 91.36 | 0.801 |
